# Common QTL Affect the Rate of Tomato Seed Germination under Different Stress and Nonstress Conditions

**DOI:** 10.1155/2007/97386

**Published:** 2008-02-10

**Authors:** Majid R. Foolad, Prakash Subbiah, Liping Zhang

**Affiliations:** Department of Horticulture and The Intercollege Graduate Degree Programs in Genetics and Plant Biology, The Pennsylvania State University, University Park, PA 16802, USA

## Abstract

The purpose of this study was to determine whether the rates of tomato seed germination under different stress and nonstress conditions were under common genetic controls by examining quantitative trait loci (QTL) affecting such traits. Seeds of BC_1_ progeny of a cross between a slow-germinating tomato breeding line and a rapid-germinating tomato wild accession were evaluated for germination under nonstress as well as cold, salt, and drought stress conditions. In each treatment, the most rapidly-germinating seeds were selected, grown to maturity, and subjected to molecular marker analysis. A selective genotyping approach detected between 6 and 9 QTL affecting germination rate under each of the four conditions, with a total of 14 QTL identified. Ten QTL affected germination rate under 2 or 3 conditions, which were considered germination-related common QTL. Four QTL affected germination rate only in one treatment, which were considered germination-related, condition-specific QTL . The results indicated that mostly the same QTL affected seed germination under different stress and nonstress conditions, supporting a previous suggestion that similar physiological mechanisms contribute to rapid seed germination under different conditions. Marker-assisted selection for the common QTL may result in progeny with rapid seed germinability under different conditions.

## 1. INTRODUCTION

The ability of the seed to germinate rapidly and uniformly under different environmental conditions is a desirable characteristic for most crop plants, including tomato, *Solanum lycopersicum* L. Seed germination is particularly important if the target environment is less than optimal during germination. Unfavorable conditions may lead to decreased rate and final percentage of seed germination, which may result in poor stand establishment and low crop yield. Under optimal germination conditions (e.g., *T* = 20–25°C and external water potential of approximately 0 kPa), most tomato seeds germinate within 2–5 days. Under stress conditions, such as extreme temperatures, high soil salinity, and water deficit, however, germination is delayed or completely inhibited depending on the intensity and duration of stress as well as genetic background of the seed. In some tomato-growing areas, the crop is established by sowing seeds directly into the field instead of using transplants. Presence of environmental stresses, however, restricts establishment of direct-seeded crops. Most commercial cultivars of tomato are sensitive to environmental stresses during seed germination and early seedling growth [1–3]. Such sensitivity renders limitations in tomato production in stress environments. Chilling sensitivity during seed germination, for example, precludes early seeding of tomatoes in the field in temperate regions and necessitates expensive heating for greenhouse production of transplants. Similarly, salt- or drought-stress sensitivity during seed germination restricts establishment of direct-seeded crops in agricultural lands affected by salt and/or water stress.

Genetic variation exists within tomato *Solanum* species for rapid seed germination under stress conditions [2, 4–6]. Such variation is potentially useful for development of cultivars with improved germination ability under stress conditions. Breeding for stress tolerance, however, requires knowledge of the genetic control of stress tolerance and of the relationships among tolerances to different stresses. Previous investigations indicated that the ability of tomato seed to germinate rapidly under stress conditions, such as high or low temperatures and salt or drought stress, was genetically controlled [3–5, 7, 8]. Furthermore, a few studies demonstrated that in tomato selection for rapid seed germination under one stress condition (e.g., cold, salt, or drought) resulted in progeny with improved germination under different stress conditions [8, 9], suggesting presence of genetic relationships among tolerances to different stresses. Furthermore, these studies indicated that selection for rapid germination under stress condition resulted in progeny with improved germination under nonstress condition. Results of these studies supported a previous suggestion that similar physiological mechanisms may control the rate of seed germination under different environmental conditions [10]. However, for scientific reasons as well as practical purposes, it is important to determine whether the same or different genes control the rate of tomato seed germination under different stress and nonstress conditions.

Tomato seed germination under different conditions exhibits continuous distributions, typical of quantitative traits [3, 7]. During the past several decades, biometrical genetic models have facilitated characterization of genetic controls of quantitative traits, including seed-related characteristics. Such models, however, have not been adequate for determining the number and chromosomal location of genes controlling quantitative traits or examining the basis of genetic relationships among traits at the molecular level. Molecular marker technology, on the other hand, has provided more accurate methods of investigating genetic controls of quantitative traits and discerning genetic relationships among traits. The goal of the present study is to determine whether the same or different quantitative trait loci (QTL) control the rate of tomato seed germination under different stress (cold, salt, and drought) and nonstress conditions by identifying and comparing QTL affecting such traits.

An effective approach to identifying genetic linkage between marker loci and QTL is trait-based marker analysis [11, 12], also know as selective genotyping [13, 14] or distributional extreme analysis [15]. The basis for this technique is that allele frequencies of genes (or QTL) affecting a trait are expected to change in response to directional selection for the trait. Selection would result in an increase in frequency of favorable alleles in the high class (e.g., fast germinators) and a decrease in the frequency of favorable alleles in the low class (e.g., slow germinators). For simply inherited traits (e.g., single-gene traits), such a change in allele frequency can easily be monitored in subsequent generations of selection. For quantitative traits, on the other hand, changes in QTL allele frequencies cannot be determined because QTL genotypes are not known. However, if some marker loci are associated with the segregating QTL (due to pleiotropic effect or physical linkage), the marker allele frequencies will also change (via “hitchhiking”) in response to selection. Any significant change in marker allele frequencies in response to selection, therefore, can be attributed to association of marker loci with QTL(s) affecting the trait under selection [11–14, 16]. In a trait-based marker analysis, marker-QTL associations can be identified either by conducting a bidirectional selection, where selection is made for both high and low classes of a response distribution [17, 18], or by conducting a unidirectional selection, where selection is made only for a high or a low class [12]. In the former case, marker-QTL associations are determined by testing the statistical significance of the marker allele frequency differences between the two extreme classes. In the latter scheme, marker-QTL associations can be determined by testing the difference between marker allele frequencies in the selected class and those in a nonselected population of the same cross. In the present study, a unidirectional selective genotyping approach was employed to identify and compare QTL contributing to rapid seed germination under nonstress as well as cold-, salt-, and drought-stress conditions.

## 2. MATERIALS AND METHODS

### 2.1. Plant materials

The tomato breeding line NC84173 was hybridized (as pistillate parent) with a fast germinating accession (LA722) of tomato wild species *S. pimpinellifolium* *L.* and F_1_ progeny was produced. NC84173 is a horticulturally superior advanced tomato breeding line (PVP) that is sensitive to cold, salt, and drought stress during seed germination and LA722 is a self-compatible accession that germinates rapidly under most conditions, including nonstress and cold, salt, and drought stress. Original seed of NC84173 and LA722 were obtained from RG Gardner, North Carolina State University (Fletcher, NC, USA) and CM Rick Tomato Genetics Resource Center, University of California (Davis, Calif, USA), respectively. A single F_1_ plant was used as pollen parent to hybridize plants of NC84173 to produce BC_1_ seed. The BC_1_ population was used for trait evaluation, genetic mapping, and identification QTL.

### 2.2. Germination evaluation of the parents and BC_1_ population

Sterile germination media, containing either 0.8% w/v agar (for nonstress as well as cold- and salt-stress treatments) or 0.3% w/v Phytagel (for drought-stress treatment), were prepared. For the drought treatment, Phytagel (Sigma-Aldrich, Miss, USA) was used as a gelling agent as agar does not gel with drought agent polyethyleneglycol (PEG). The germination medium for the salt treatment also included 150 mM NaCl + 15 mM CaCl_2_ and that for the drought treatment included 14% PEG. Germination media were prepared in 15-cm round Petri plates. The water potentials (*ψ*) of the treatment media were −30, −30, −690, and −680 kPa for the control, cold, salt, and drought treatments, respectively, as measured on a Wescor-5100 vapor pressure osmometer (Wescor, Logan, Utah, USA). Seeds of parental lines (NC84173 and LA722) and BC_1_ population were surface-sterilized with 0.5% NaOCl solution for 10 minutes, rinsed with sterile, distilled water, and briefly blotted. For each of the control, cold, salt, and drought treatments 1000 seeds of BC_1_ generation and 192 seeds of each of the parental lines were sown on germination media under aseptic conditions. Each Petri plate contained 64 seeds and was considered as one replicate. Petri plates were placed in a completely randomized design (CRD) in incubators maintained in dark at either 20 ± 0.5°C (control, salt, and drought treatments) or 11 ± 0.5°C (cold treatment). Germination responses were scored visually as radicle protrusion at 8-hour intervals for 37 consecutive days. To estimate mean germination time, germination distributions of the parental lines and BC_1_ population in the four treatments were subjected to survival analysis, [19] and the time, in days, to 50% germination was obtained for each replicate and averaged over replicates.

### 2.3. Selection for rapid seed germination under different conditions in BC_1_ generation

In each of the control, cold, salt, and drought-stress treatments the 30 most rapidly germinating BC_1_ seeds (the first 3% germinated) were selected (hereafter referred to as “selected classes”). Selected seedlings from the different treatments were transplanted into greenhouse seedling trays and subsequently into a field, where they were grown to maturity and self-pollinated to produce BC_1_S_1_ progeny seed. The BC_1_S_1_ progeny were examined for rate of seed germination under different conditions, as described elsewhere [8].

### 2.4. Marker genotyping of the selected BC_1_ plants

Leaf tissue from each of the 120 selected BC_1_ plants was collected for DNA isolation and marker analysis. Nuclear DNA was extracted using standard protocols for tomato [20]. DNAs were treated with RNAse and digested with 5 restriction enzymes, including *Dra*I, *Eco*RI, *Eco*RV, *Hind*III, and *Xba*I according to the manufacturer's instructions and subjected to gel electrophoresis. Genomic blots were prepared and hybridized with 119 DNA probes, which previously were determined to detect polymorphism between the two parents [21], including 112 random genomic or cDNA clones, obtained from Steven Tanksley, Cornell University, Ithaca (NY, USA), and 7 germination related cDNA clones, obtained from Kent Bradford, University of California (Davis, Calif, USA). The RFLP markers were chosen so to have a good coverage of the 12 tomato chromosomes. Except for chromosomes 9 and 11, for which limited RFLP polymorphism was identified between the two parents, at least 9 RFLP markers were used for each chromosome. Probes were labeled with ^32^P-dCTP by primer extension [22]. Agarose gel electrophoresis, Southern blotting, hybridizations, and autoradiography were as described elsewhere [17].

### 2.5. Marker genotyping of a nonselected BC_1_ population

A nonselected BC_1_ population (*N* = 119) of the same cross (NC84173 ×LA722) was previously genotyped with 151 RFLP markers, including the 115 markers used in the present study, and a genetic linkage map was developed [21]. In the present study, marker data from the previous mapping population were used to calculate allele frequencies in a nonselected (random) BC_1_ population, which then were used to calculate differences in marker allele frequencies between selected and nonselected populations and identify QTL, as described below.

### 2.6. Statistical analyses and identification of QTL

A selective genotyping approach was employed to identify QTL affecting germination rate under control, cold, salt, and drought conditions. The genotypes of the 30 selected BC_1_ plants from each of the four selection treatments (control, cold, salt, and drought) were determined for the 119 RFLP markers. Using the genotypic numbers obtained for the 119 RFLP markers, marker allele frequencies were determined for each of the four selected classes. The variance of allele frequency for each marker was calculated as a binomial variance ($s_{q}^{2}=pq/2N$), where *p* and *q* are the corresponding allele frequencies at a given marker locus and *N* is the number of individuals genotyped at that locus [23]. Similar marker analyses were conducted on the nonselected BC_1_ population [21] and allele frequencies for the 115 markers were calculated.

Marker allele frequency differences between each of the selected control, cold, salt, and drought classes and the nonselected BC_1_ population (*q_S_-q_NS_*) were determined, where *q_S_* is the frequency of the *i*th allele at the *k*th marker locus in each of the selected classes (*N* = 30) and *q_NS_* is the frequency of the *i*th allele at the *k*th marker locus in the nonselected population (*N* = 119). Allele frequency differences between the selected classes and the nonselected population were considered significant when *q_S_-q_NS_* ≥2*σq*, where **σ*q* = *(p_S_q_NS_/2N_S_* *+ p_NS_q_NS_/2N_NS_)^1/2^* is the standard error of the difference between marker allele frequencies, *N_S_* is the number of BC_1_ progeny in each selected class, and *N_NS_* is the number of individuals in the nonselected BC_1_ population. This test provides a confidence of more than 95% on the identified QTL [11, 14, 23, 24]. At each marker locus, significant allele frequency difference between a selected class and the nonselected population was inferred as an association of the marker locus with a major QTL [12, 13, 17, 25]. However, in cases where *q_S_-q_NS_* was smaller than *2*σ*q* but greater than 1**σ*q,* the marker was judged to be associated with a QTL with minor effects.

### 2.7. Estimation of QTL effects

While selective genotyping is more powerful than standard marker-based (interval mapping) analysis in detecting linkage between markers and QTL (primarily because of the use of large population), it is less efficient in determining QTL effects. Individuals in the high class tend to have a large number of positive QTL alleles and individuals in the low class tend to have a large number of negative QTL alleles, and there is a deficiency of individuals with a mixture of positive and negative alleles in the subpopulations being analyzed (this is particularly true for traits with high heritability). This deficiency hampers the ability to measure the effect of individual QTL using traditional analysis of variance. However, the approximate effects of QTL can be estimated using an equation that relates the change in marker allele frequencies due to selection with the QTL effects (described below). This equation assumes no recombination between the marker locus and the QTL. When this assumption is met, the larger the effects of the QTL, the greater would be the difference in marker allele frequency in response to selection. However, if recombination occurs between the marker and the QTL, the effect of the QTL would be underestimated.

Falconer [23] provided an expression relating the selection intensity, *i*, with the coefficient of selection, *s*, acting on an individual gene (or QTL) as follow:

(1) s=iD,

where *D = 2 d/* **σ** *_P_* is the standardized effect of the QTL (in phenotypic standard deviation unit, **σ** *_P_*) and *d* is the phenotypic difference between the two homozygotes at the QTL. With further substitution for *s* and assuming no recombination between the marker and the QTL, the standardized effect of a QTL, as a function of selection intensity and the difference in allele frequencies at a linked marker resulting from a one-step directional selection in a BC_1_ population, can be estimated as

(2) $$D=\frac{2\delta q}{\left[iq(1-q)\right]},$$

where **δ**_*q*_ is the difference in marker allele frequencies between a selected class and the nonselected population (i.e., *q_S_-q_NS_*), *i* is the selection intensity (i.e., standardized selection differential), and *q* is the allele frequency at the QTL-linked marker locus in the nonselected population. Using this expression, the approximate standardized effects of the marker-linked QTL (i.e., the difference between the two homozygotes at a QTL in standard unit) was estimated. It should be noted that the calculated values are considered minimum effects of QTL due to likely recombinations between markers and QTL.

## 3. RESULTS

### 3.1. Germination rates of the parental lines and BC_1_ progeny

Seed of the wild accession LA722 germinated significantly more rapidly than seed of the breeding line NC84173 under nonstress (control) as well as cold, salt, and drought stress conditions; the difference between the two parents, however, was greater under stress than nonstress conditions (Table 1). This is consistent with previous reports on germination rates of these and other lines [2, 6]. Seed of the BC_1_ population germinated intermediate between the two parental lines, indicating the inheritance of rapid germination from LA722 to the progeny (Table 1).

### 3.2. Map construction

**Figure 1:**
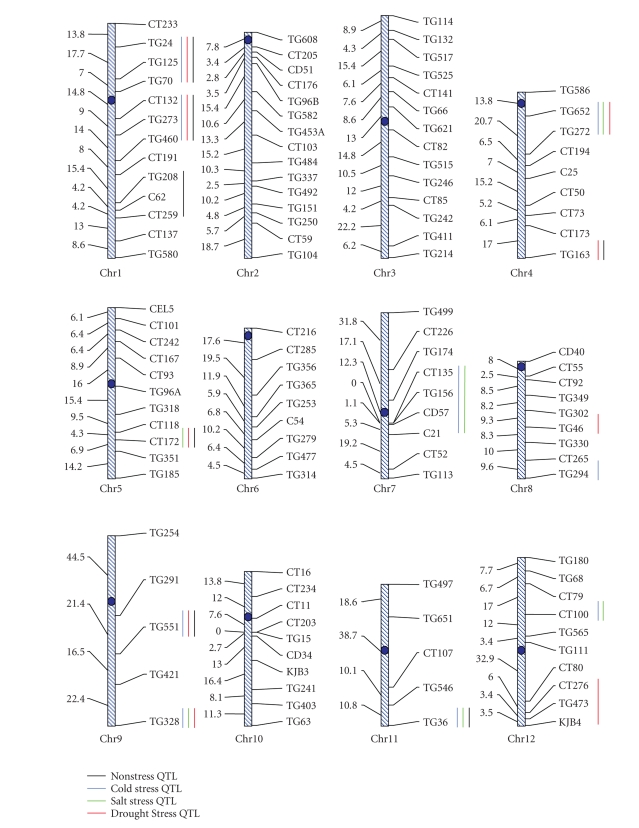
An RFLP linkage map of the 12 tomato chromosomes constructed based on a BC_1_ population of a cross between *Lycopersicon esculentum* (NC84173) and *L. pimpinelliforlium* (LA722). The names of the markers are shown at the *right* of the chromosomes. The map position of all markers is shown at the *left* of the chromosomes (in centiMorgan based on the Kosambi function). The black, blue, green, and red vertical lines at the *right* of the chromosomes indicate the approximate locations of QTL for germination rate under control (nonstress) and cold-, salt-, and drought-stress conditions, respectively.

Using a nonselected BC_1_ population (*N* = 119 individuals) of the same cross from a previous study [21] and the 119 RFLP markers scored in both the nonselected population and the selected classes, a genetic linkage map was constructed using computer program MAPMAKER v. 3.0 [26]. The procedure for map construction was similar to previous studies [21, 27]. This map covered 1172 cM of the 12 tomato chromosomes with 9.7 cM distance between adjacent markers (see Figure 1), as estimated based on Kosambi function [28].

### 3.3. Identification of QTL for germination under different conditions

*QTL for germination under nonstress (control) conditions*. Four major QTL (on chromosomes 1, 4, and 9) and 3 minor QTL (on chromosomes 1, 5, and 11) were identified for germination under nonstress conditions (see Table 2, Figure 1). All QTL for rapid seed germination under nonstress conditions were contributed from the rapid-germinating wild donor parent, LA722. The standardized effects (*D*) of the QTL ranged from 0.38 to 0.87 phenotypic standard deviation.

*QTL for germination under cold stress conditions*. Three major QTL (on chromosomes 4, 8, and 9) and 6 minor QTL (on chromosomes 1, 7, 9, 11, and 12) were identified for germination under cold stress (see Table 2, Figure 1). For all QTL, but two on chromosomes 4 and 8, the positive alleles were contributed from LA722 (Table 2). The standardized effects (*D*) of the identified QTL ranged from 0.31 to 0.69 phenotypic standard deviation.

*QTL for germination under salt stress conditions*. Four major QTL (on chromosomes 5, 7, 9, and 11) and two minor QTL (on chromosomes 4 and 12) were identified for germination under salt stress (see Table 2, Figure 1). All QTL but one on chromosome 4 were contributed from LA722 (Table 2). Furthermore, the QTL that was contributed from the slow-germinating recurrent parent had smaller effects than those contributed from the donor parent. The standardized effects (*D*) of the identified QTL ranged from 0.45 to 1.01 phenotypic standard deviation.

*QTL for germination under drought stress conditions*. Two major QTL (on chromosomes 8 and 9) and seven minor QTL (on chromosomes 1, 4, 5, 9, and 12) were identified for germination under drought stress (see Table 2, Figure 1). For all QTL except three on chromosomes 4, 8, and 12, the positive alleles were contributed from LA722 (see Table 2, Figure 1). Furthermore, QTL that were contributed from LA722 had generally larger effects than those contributed from the slow-germinating cultivated parent, NC84173. The standardized effects (*D*) of the identified QTL ranged from 0.33 to 1.02 phenotypic standard deviation.

### 3.4. Comparison of QTL across treatments

A total of 14 QTL were identified with significant effects on germination rate under one or more conditions. Of these, 4 QTL (29%) affected only one trait and 10 QTL (71%) affected 2 or 3 traits. The QTL affecting one trait included one on chromosome 1 affecting germination under control (nonstress) condition, two on chromosome 8 affecting germination under cold or drought stress, and one on chromosome 12 affecting germination under drought stress. Three QTL affected 2 traits, including one on chromosome 4 affecting germination under drought and nonstress and one on each of chromosomes 7 and 12 affecting germination under cold and salt stress. Seven QTL (50% of the total) affected germination rate under three different conditions, including 3 on chromosomes 1 and 9 affecting germination under cold, drought, and control conditions, 2 on chromosomes 4 and 9 affecting germination under cold, salt, and drought conditions, one on chromosome 5 affecting germination under salt, drought, and control conditions, and one on chromosome 11 affecting germination under cold, salt, and control conditions (see Table 2, Figure 1). Ten of the 14 QTL (71%) were contributed from the fast germinating donor parent (LA722) whereas four from NC84173.

### 3.5. Germination response of the BC_1_S_1_ progeny

Evaluation of the germination response of the BC_1_S_1_ progeny indicated that selection for rapid seed germination under any of the four conditions in the BC_1_ generation resulted in progeny with improved germination under all four conditions (Table 3; [8]). The improvement in germination rate of the selected BC_1_S_1_ progeny was significant when compared to germination rate of the nonselected BC_1_S_1_ progeny (Table 3).

## 4. DISCUSSION

### 4.1. Number, genetic effect, and location of QTL

The power of selective genotyping in detecting QTL depends on several factors, including heritability (*h^2^*) of the trait, gene action, the type of mapping population, the intensity of selection, the number and individual effects of QTL, the extent of marker coverage, and the distance between marker loci and QTL affecting the trait [11, 13, 14, 17, 23]. In the present study, the use of a rather large population (*N* = 1000), an intensive selection (*p* = 3%), and a relatively good marker coverage provided sufficient power to detect many putative QTL. For each trait, between 2 and 4 major QTL (i.e., *dq* ≥ *2*σ*q*) and 2–7 minor QTL (1**σ*q* ≤ *dq* < 2**σ*q*) were detected. However, due to moderate heritabilities of these traits (*h^2^* = 0.20–0.75; [8, 9]) and because trait evaluation was conducted in BC_1_ generation, where donor-parent QTL with recessive effects would not be detected, it is likely that some QTL remained undetected. Therefore, the QTL identified in this study for each trait should be considered the minimum number of QTL affecting the trait. Additional QTL may be identified if advanced populations such as recombinant inbred lines (RILs) or backcross inbred lines (BILs) are used. Also, the calculated standardized effects of QTL (*D*; Table 2) should be considered lowest estimates as the assumption of no recombination between markers and QTL may not be valid in all cases. However, in a selective genotyping approach, the accuracy of QTL-effect estimation can be greatly improved by using higher density map, larger size population, and more advanced generation. This is also true in case of interval mapping.

The intervals for a few QTL, including those identified on chromosomes 1 and 4, were rather large (20–25 cM; see Figure 1). Whether each of these genomic regions contains one QTL or multiple linked QTL could not be determined in this study. Similar to F_2_ generation, in BC_1_ generation of a cross between two inbred lines linkage disequilibrium is large and consequently loosely-linked flanking markers may also show association with QTL and it may not be possible to determine the exact position of QTL [29]. This is a general concern when using early segregating populations for genetic mapping, irrespective of employing a trait-based (selective genotyping) or a marker-based (interval mapping) analysis. However, the use of advanced segregating populations, such as RILs or BILs in which linkage disequilibrium is reduced, large size populations, and composite interval mapping approach [30, 31] is expected to provide for a better delineation of QTL position.

### 4.2. Comparison of QTL with those previously identified

In two previous studies, using traditional interval mapping approach and backcross (BC_1_S_1_) populations of the same cross as in this study, QTL were identified for rapid seed germination under cold stress (on chromosome 1 and 4; [32]) and salt stress (on chromosomes 1, 9, and 12; [33]). The present study detected all of those QTL except one QTL on chromosome 1 for germination under salt stress (see Figure 1). This high level of consistency between the previous and present studies suggests the efficacy of the screening methods and the reliability of the identified QTL. The present study also identified a few additional QTL for these traits indicating a greater power of selective genotyping in detecting QTL, primarily due to the use of large populations and intense selections.

### 4.3. Comparison of QTL affecting germination rate under different conditions

Ten of the 14 identified QTL (71%) affected germination rate under 2 or 3 conditions, of which 7 (50% of the total) affected germination rate under three different conditions (Table 2, see Figure 1). This finding indicates the presence of germination-related common QTL/genes in tomato which affect germination rate under different conditions. The presence of common QTL suggests presence of genetic relationships between the ability to germinate rapidly under different conditions and the expectation that selection and improvement of seed germination under one condition would lead to progeny with improved germination under other conditions. This QTL-based prediction is in fact in agreement with previous findings of presence of phenotypic and genetic correlations between the rates of tomato seed germination under different conditions and with results of selection experiments [6, 8, 9]. It is therefore concluded that in tomato, the ability of the seed to geminate rapidly under different stress and nonstress conditions is at least partially controlled by the same QTL. However, whether the effects of common QTL were due to pleiotropic effects of the same genes, physical linkage of different genes, or a combination of both could not be determined in the present study. The finding of common QTL, nonetheless, has fundamental and practical implications, as discussed below.

In comparison, only 4 of the 14 QTL (29%) affected germination only in one treatment (see Table 2, Figure 1). The identification of these QTL suggests presence of genes which affect germination rate only under specific environmental conditions. Interestingly, 3 of these 4 QTL, those on chromosomes 8 and 12, had the positive QTL alleles (for rapid germination) from the slow-germinating recurrent parent. However, the paucity of such QTL and the preponderance of QTL with common effects indicate the significance of genetic factors which affect tomato seed germination under different conditions. This genetic finding is in agreement with previous physiological studies of tomato seed germination under different conditions, as discussed below.

### 4.4. Physiological mechanisms of germination under different conditions

Excessive salt in the germination medium depresses water potential, making water less available to the seed, which may reduce the rate or completely inhibit seed germination. Low rate of germination under salt stress could be due to osmotic and/or ionic effects of the saline medium. The available evidence, however, suggests that low water potential of the germination medium rather than its ion toxicity effects is the major limiting factor to germination under salt stress in different crop species, including tomato [10, 34–36]. Furthermore, a more recent detailed investigation of tomato seed germination under different stress conditions, using various ionic and nonionic germination media with identical osmotic potential, confirmed that germination rate was mainly affected by osmotic rather than ionic effects of the medium [37].

Under drought stress, reduced water potential of the germination medium is the cause of slow seed germination [10]. This is similar to the condition under salt stress. Therefore, it is not unexpected that seeds that withstand the low water potential and germinate rapidly under drought stress also germinate rapidly under salt stress, and vice versa. This is also in agreement with the finding of a previous study that indicated the presence of correlation (*r* = 0.82, *P* < .01) between germination rate under salt and drought stress in tomato [8]. Therefore, it is likely that similar or identical genes (and physiological mechanisms) may control the rate of tomato seed germination under salt and drought stress. Support for this suggestion is the observation of a significant improvement in germination rate under drought stress in response to selection for rapid seed germination under salt stress, and vice versa [8].

Under cold stress, the delay in seed germination could also be due to water stress as low temperature does affect water status of the cell [38]. However, whether genetic and physiological processes which impart rapid seed germination under salt and/or drought stress also could facilitate rapid germination under cold stress is unknown. In the present study, however, the finding that almost all QTL for germination under cold stress colocalized with QTL for germination under salt and/or drought stress suggests that the same genes (or physiological mechanisms) may contribute to rapid seed germination under these three conditions. This suggestion is consistent with the finding that selection for rapid seed germination under salt or drought stress resulted in progeny with improved germination rate under cold stress, and vice versa [8]. However, isolation, characterization, and comparison of functional genes, which facilitate rapid seed germination under the various conditions, are necessary in order to determine the exact genetic relationships among these traits. Nonetheless, results of the present study suggest presence of genetic relationships in the ability to germinate rapidly under different stress conditions and that MAS for common QTL would lead to progeny with improved germination rate under all these conditions.

In the present study, 7 major or minor QTL were identified affecting germination rate under nonstress (control) conditions (see Table 2; Figure 1). Of these only one QTL (located on the lower part of chromosome 1) affected germination only under the nonstress condition whereas the rest affected germination under three (5 QTL) or two conditions (1 QTL). Furthermore, as determined in this study, selection for rapid seed germination under nonstress condition resulted in progeny that germinated significantly faster than nonselected progeny under both nonstress and stress (cold, salt, and drought) conditions (Table 3). These findings suggest that at least some of the genes or physiological mechanisms which facilitate rapid seed germination under nonstress conditions also contribute to rapid germination under stress conditions. Furthermore, the previous findings that selection under stress conditions resulted in progeny with faster germination ability under nonstress condition [8, 9] suggest that genetic controls facilitating rapid seed germination under stress conditions do not have undesirable effects on performance in the absence of stress. These genetic findings are in agreement with results of physiological studies of tomato seed germination, which suggested involvement of common physiological mechanisms contributing to rapid germination under different conditions [10]. It appears that seeds that have the desirable genetic components for rapid germination tend to germinate rapidly under a wide range of environmental conditions. It is, therefore, likely that MAS based on germination-related common QTL would result in progeny with improved germination under both stress and nonstress conditions.

### 4.5. QTL with effects in opposite direction to the parental phenotypes

For majority (71%) of the identified QTL, the positive alleles were contributed from the rapid germinating donor parent, LA722. This was not surprising because of the significant differences between the two parents in germination rate in all four treatments (Table 1). However, t QTL (from a total of 14), located on chromosomes 4, 8, and 12, were identified for which the slow germinating parent (NC84173) contributed the positive alleles for rapid germination (Table 2). Although the number of positive QTL contributed from NC84173 was much smaller than that from LA722, the results suggested the presence of potentially useful QTL for rapid seed germination in the slow germinating parent. Such finding is not uncommon and has been reported in the literature for many other traits in various plant species. The identification of QTL with effects in opposite directions to the parental phenotypes demonstrates the ability of marker analysis to uncover cryptic genetic variation that otherwise would have been masked by the large phenotypic differences between the parents. Furthermore, the presence of such QTL suggests the likelihood of recovering transgressive variants in segregating populations derived from crosses between contrasting parents.

## 5. CONCLUSION

The present study identified between 6 and 9 QTL for each of the four germination traits. While four QTL were identified, each affecting only one trait, the majority of the QTL (71%) were common across the treatments and affected rate of seed germination under two or three conditions. The identification of germination-related common QTL indicates that the rate of tomato seed germination under different conditions is at least partially under the same genetic controls, confirming previous reports of presence of correlations among the rate of tomato seed germination under different conditions. It further suggests that similar physiological mechanism(s) may facilitate rapid seed germination under different conditions, congruent with the findings of previous physiological studies of tomato seed germination. It is, therefore, expected that tomato seed germinability under different conditions can be improved by marker-assisted selection for common QTL. In this regards, the seven QTL on chromosomes 1, 4, 9, and 11 which were identified with effects on germination rate under three conditions (Table 2) should be the most useful QTL for MAS improvement of tomato seed germinability under different conditions.

## Figures and Tables

**Table 1:** Mean days to 50% germination (±SE) for the parental lines and the BC_1_ population of an interspecific cross between a slow germinating tomato (*Lycopersicon* *esculentum*) breeding line (NC84173) and a fast germinating *L. pimpinellifolium* accession (LA722) in the control (nonstress) and cold-, salt-, and drought-stress treatments.

Genotype	*n* (per treatment)	Control	Cold stress	Salt stress	Drought stress
P_1_ (NC84173)	512	3.2±0.1	14.9±0.6	13.6±0.7	12.5±0.6
P_2_ (LA722)	512	1.7±0.1	6.5±0.3	4.2±0.2	3.2±0.2
BC_1_ [P_1_(P_1_ ×P_2_)]	1000	2.4±0.2	9.8±0.9	10.6±1.1	8.0±0.7

**Table 2:** Chromosomal locations of QTL associated with the rate of tomato seed germination under nonstress (control) and cold-, salt- and drought-stress conditions.

		m20Chr.													
		1	1	1	4	4	5	7	8	8	9	9	11	12	12
Marker		TG24-TG70	CT132-TG460	TG208-CT259	TG652-TG272	TG163	CS172	CT135-C21	TG46	TG294	TG551	TG328	TG36	CT100	CT276-KJB4
inteval															

Allele freq.		0.25	0.28	0.28	0.27	0.26	0.25	0.22	0.26	0.29	0.24	0.21	0.19	0.29	0.24
nonsel. pop.															

Control	*q_CS_*	0.37	0.42	0.43	0.28	0.45	0.33	0.18	0.23	0.30	0.38	0.23	0.30	0.28	0.27
(CT)	*q_CS_*-*q_NS_*	0.12*	0.14**	0.15**	0.01	0.19**	0.08*	−0.04	−0.03	0.01	0.14**	0.02	0.11*	−0.01	0.03
	*σ* *q* ^(a)^	0.07	0.07	0.07	0.07	0.07	0.07	0.06	0.06	0.07	0.07	0.06	0.06	0.07	0.06
	*D* ^(b)^	**0.56**	**0.61**	**0.66**		**0.87**	**0.38**				**0.68**		**0.63**		

Cold	*q_SS_*	0.33	0.35	0.30	0.13	0.22	0.28	0.32	0.27	0.15	0.38	0.30	0.32	0.40	0.22
stress (CS)	*q_SS_-q_NS_*	0.08*	0.07*	0.02	−0.14**	−0.04	0.03	0.10*	0.01	−0.14**	0.14**	0.9*	0.12*	0.11*	−0.02*
	*σ* *q*	0.07	0.07	0.07	0.05	0.06	0.06	0.07	0.06	0.05	0.07	0.07	0.07	0.07	0.06
	*D*	**0.38**	**0.31**		−**0.63**			**0.51**		−**0.60**	**0.68**	**0.48**	**0.69**	**0.47**	

Salt stress	*q_DS_*	0.24	0.25	0.34	0.17	0.22	0.42	0.38	0.23	0.27	0.27	0.40	0.33	0.40	0.29
(SS)	*q_DS_-q_NS_*	−0.01	−0.03	0.06	−0.10*	−0.04	0.17**	0.16**	−0.03	−0.02	0.03	0.19**	0.14**	0.11*	0.05
	*σ* *q*	0.07	0.07	0.07	0.06	0.06	0.07	0.07	0.06	0.06	0.06	0.07	0.07	0.07	0.07
	*D*				**−0.45**		**0.80**	**0.82**				**1.01**	**0.80**	**0.47**	

Drought	*q_CT_*	0.37	0.39	0.31	0.18	0.37	0.32	0.23	0.12	0.27	0.45	0.28	0.20	0.27	0.13
stress (DS)	*q_CT_-q_NS_*	0.12*	0.11*	0.04	−0.09*	0.11*	0.07*	0.01	−0.14**	−0.02	0.21**	0.07*	0.01	−0.02	−0.11*
	*σ* *q*	0.07	0.07	0.07	0.06	0.07	0.07	0.06	0.05	0.06	0.07	0.06	0.06	0.06	0.05
	*D*	**0.56**	**0.48**		−**0.40**	**0.50**	**0.33**		−**0.64**		**1.02**	**0.37**			−**0.53**

^(a)^ Standard error of the difference between marker allele frequencies of the selected and nonselected populations; ^(b)^Approximate standardized effects of QTL in phenotypic standard deviation unit; ${}^{*},{}^{**}$ Marker allele frequency difference greater than 1*σ* *_q_* or 2*σ* *_q_*, respectively.

**Table 3:** Meandays to 50% germination (± SE) of the selected BC_1_ S_1_ progeny and the selection response (percentage gain relative to the nonselected BC_1_ S_1_ population) in the control (nonstress) and cold-, salt- and drought-stress treatments. The mean germination for the nonselected BC_1_ S_1_ progeny in different treatments are also shown (data partially taken from Foolad et al. 2003).

Treatment during Progeny Evaluation								
Treatment	Control		Cold Stress		Salt stress		Drought stress	
during	Mean	Response^1^	Mean	Response	Mean	Response	Mean	Response
selection	(Days)	(%)	(Days)	(%)	(Days)	(%)	(Days)	(%)
Cold stress	1.8±0.2	9.5*	6.7±1.9	15.5**	5.8±1.0	16.2**	5.3±1.0	18.3**
Salt stress	1.8±0.2	8.6*	6.4±1.0	19.6**	5.4±0.7	22.2**	5.2±0.8	18.5**
Drought stress	1.8±0.2	8.0*	6.9±1.7	13.5**	5.0±0.8	28.1**	5.2±1.3	19.6**
Nonstress (control)	1.8±0.2	8.7*	6.2±1.7	20.9**	4.6±0.9	33.5**	5.3±1.1	17.8**
Nonsel. BC_1_ S_1_	2.0±0.1		7.9±0.9		6.9±1.0		6.4±0.8	

*,** Significant at the 5 and 1% probability levels, respectively. ^1^Response to selection was measured as the percentage difference in germination mean between the selected and nonselected BC_1_ S_1_ progenies.
